# Assessment of *cyclooxygenase-1* and *2* gene expression levels in chronic autoimmune thyroiditis, papillary thyroid carcinoma and nontoxic nodular goitre

**DOI:** 10.1186/s13044-014-0010-2

**Published:** 2014-12-17

**Authors:** Kinga Krawczyk-Rusiecka, Katarzyna Wojciechowska-Durczynska, Anna Cyniak-Magierska, Arkadiusz Zygmunt, Andrzej Lewinski

**Affiliations:** Department of Endocrinology and Metabolic Diseases, Medical University of Lodz, Rzgowska St. No. 281/289, 93-338 Lodz, Poland; Polish Mother’s Memorial Hospital – Research Institute, Lodz, Poland

**Keywords:** COX-2 expression, PTC, HT, FNAB washouts

## Abstract

**Introduction:**

The cyclooxygenases are a group of enzymes catalyzing the formation of prostaglandins from arachidonic acid. Cyclooxygenase-1 (COX-1) is a constitutive form, thought to be a “housekeeping gene”, with constant levels of expression in most tissues. *COX-1* expression in the thyroid gland, except for medullary thyroid carcinoma, has not been a subject of much interest. Cyclooxygenase-2 (COX-2) can be expressed in response to various stimuli, such as mitogens, hormones, cytokines, growth factors. The product of *COX-2* activity has been implicated in carcinogenesis.

Recent studies have shown that up-regulation of COX-2 is associated with numerous neoplasms. Hereby, we present a study analysing *COX-1* and *COX-2* expression in papillary thyroid carcinoma (PTC), Hashimoto thyroiditis (HT) and nontoxic nodular goitre (NNG) in fine needle aspiration biopsy (FNAB) washouts and in postoperative tissue.

**Material and methods:**

Cytological specimens from 120 patients (105 females and 15 males) have been studied, including patients with HT, PTC and NNG. Moreover, we have examined postoperative tissue specimens from 51 patients with PTC and NNG.

The methods of molecular analysis have included extraction of total RNA from FNAB cytological material and postoperative tissues, spectrophotometric assessment of the RNA purity, cDNA synthesis in reverse transcription reaction and an analysis of genes expression data by real-time PCR.

**Results:**

The performed analysis has revealed statistically significant higher expression level of the *COX-2* gene in PTC group, in comparison with HT and NNG groups (in both cytological and postoperative material).

In PTC patients, *COX-2* gene expression levels in the material obtained by FNAB were similar to those in the postoperative thyroid tissue.

No correlations between *COX-2* gene expression level and TNM staging in PTC samples have been observed.

There were no correlations between *COX-2* expression and anti-TPO antibodies level, or patient’s sex or age in the studied groups. Also, there were no correlations of *COX-1* gene expression level among PTC, HT and NNG groups.

**Conclusions:**

Our results suggest that *COX-2* gene does not participate in the mechanisms involved in molecular association of HT with PTC. However, in case of PTC itself, it may play some role in neoplastic transformation.

## Introduction

The cyclooxygenases are a group of enzymes that catalyze the formation of prostaglandins from arachidonic acid [1]. Cyclooxygenase 1 (COX-1) is a membrane-bound enzyme present in many tissues and organs involved in the synthesis of thromboxane A2 (TXA2) and prostacyclines (PGI2). *COX-1* gene is located on chromosome 9q and encodes a 66-kDa protein; its expression is constant in most tissues and belongs to the so-called “housekeeping genes” [2]. *COX-1* gene expression in the thyroid gland (except for the medullary thyroid carcinoma [3,4] has not been a subject of interest. *COX-2* gene is located on the long arm of chromosome 1 (position lq25.2-q25.3), contains 10 exons and encodes a protein of 70 kDa. COX-2, also called an inducible isoform, is primarily identified with the body’s response to stress factors and initiation of inflammation, its expression is tightly controlled under basal conditions, increases significantly in pathological conditions, under the influence of cytokines, hormones, inflammatory mediators and mitogens. It can inhibit apoptosis and induce proliferation of tumour cells, also the ability of these cells to form metastases and invasion [5,6].

Increased COX-2 activity is associated with the occurrence of many different neoplasms such as squamous cell carcinoma of the skin, lung, colorectal, breast, prostate, pancreas, liver, stomach, skin, bladder and ovarian cancers.

*In vivo* models have shown that prostaglandins (PGs) - produced in response to the action of the enzyme COX-2 - are responsible for the stimulation of angiogenesis in tumours, and the use of COX-2 inhibitors slows down the process of neovascularization [7]. In addition, microvessel density in tumours of the colon appears to be closely related to the *COX-2* gene expression level.

In recent decades, there is an increasing incidence of thyroid cancer around the world [8]. On the other hand, the effects of COX-2 enzyme in inflammatory processes of the thyroid have recently become a subject of interest for researchers. Additionally, the relationship between Hashimoto thyroiditis and the incidence of thyroid cancer is complex and not yet fully explained [9–11]. In the present study, relative expression levels of *COX-1* and *COX-2* gene in the fine needle aspiration biopsy (FNAB) washouts and in postoperative tissue of patients with papillary thyroid carcinoma (PTC), Hashimoto thyroiditis (HT) and nontoxic nodular goitre (NNG) were evaluated. As far as we are concerned, our present study includes the largest group of HT patients extensively studied by molecular methods.

## Materials and methods

The procedures were approved by the Ethical Committee of the Medical University of Lodz (Poland). All patients were informed and agreed to participate in this study.

One hundred seventy one (171) thyroid specimens were analyzed. Cytological specimens came from 120 patients, and the tissue material - from 51 patients. There were 62 cases of papillary thyroid carcinoma (PTC), 56 cases of Hashimoto’s thyroiditis (HT) and 53 cases of nontoxic nodular goitre (NNG) collected from 28 men and 143 women. Patients ranged in age from 24 to 77 years (median: 52 years for PTC group, 49 years for HT group, 57 years for NNG group).

Each aspirate was smeared for conventional cytology, while the remaining part of aspirate was immediately washed out of the needle. The cells, obtained from the needle, were used for further investigation.

Tissue samples from the postoperative specimens of PTC and NNG were obtained. Samples of macroscopically unchanged thyroid tissue collected from patients with nodular goitre served as an internal control for Real –time experiment.

Total RNA was extracted using RNeasy Micro Kit (FNAB material) and RNeasy Midi Kit (postoperative material) (Qiagen, Hilden, Germany), based on modified Chomczynski and Sacchi’s method [12]. RNA concentration was spectrophotometrically assessed by measuring absorbance at 260 and 280 nm (NanoDrop ND-1000 Spectrophotometer, Thermo Fisher Scientific, USA).

Total RNA was reversely transcribed in a Mastercycler personal thermal cycler (Eppendorf, Hamburg, Germany) according to manufacturer’s procedures in a total volume of 50 μL (TaqMan Reverse Trancriptase Reagents, Applied Biosystems, Foster City, CA, USA).

The relative expression of *COX-2* gene was assessed using the ABI PRISM 7500 SDS Software (ABI PRISM 7500 Sequence Detection System, Applied Biosystems), according to the manufacturer’s protocol. The PCR reactions for *COX-2* gene were run with 5 μL of cDNA in a total volume of 50 μL, using TaqMan Universal PCR Master Mix (Applied Biosystems, Foster City, CA, USA) and predesigned primer/probe set (Assays-on-Demand™, Gene Expression assay, Hs 00153133_m1, Hs 00377721_m1 Applied Biosystems). Amplification reactions were done in triplicate for each examined sample.

Controls with no template cDNA were used with each assay. The reference gene was *ACTB* (Assays-on-Demand™ Gene Expression assay, Hs 99999903_m1, Applied Biosystems).

Cycling conditions were 50°C for 2 min and 95°C for 10 minutes, followed by 50 cycles at 95°C and 60°C for 1 minute. Macroscopically unchanged thyroid tissue served as a calibrator to compare the relative amount of target in different samples and to adjust for the plate-to-plate variation in amplification efficiency.

### Data and statistical analysis

Statistical analysis was performed using statistical software package Statistica 7.0. Basic measures of location (i.e. mean, median values) and dispersion [standard deviation (SD), standard error of mean (SEM)] were calculated. P values < 0.05 were considered to indicate statistical significance.

Relative gene expression is determined by comparing threshold cycle (C_t_) for gene of interest (*COX-2*) with C_t_ for the reference gene (*β-actin*) and was calculated using the ΔΔC_T_ method on an ABI PRISM® 7500 Sequence Detection System Software (Applied Biosystems, Foster City, CA, USA).

The data were statistically analyzed using a standard parametric Student’s t-test, followed by non-parametric Kruskal-Wallis one-way analysis of variance by ranks, Tukey range test and non-parametric Mann–Whitney U test.

## Results

**Figure 1:**
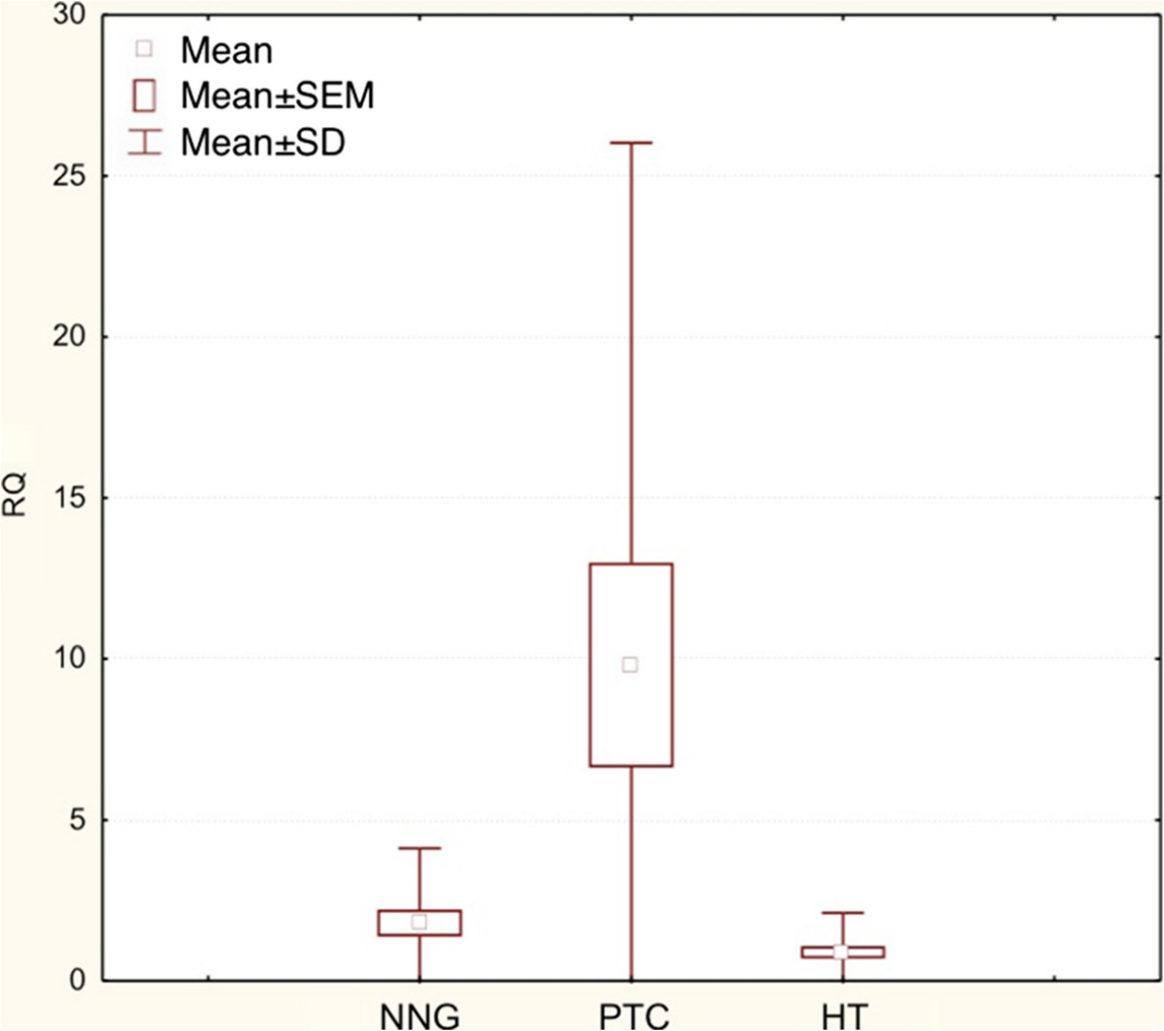
**Box-and-whisker plots representing the** ***COX-2*** **gene expression by quantitative RT-PCR in NNG, PTC and HT groups (FNAB).** The results are calculated as RQ values. Whiskers represent mean ± SD (standard deviation) for particular groups. Boxes represent mean ± SEM (standard error of mean). The results were statistically analyzed by Kruskal-Wallis test (p < 0.0001).

**Figure 2:**
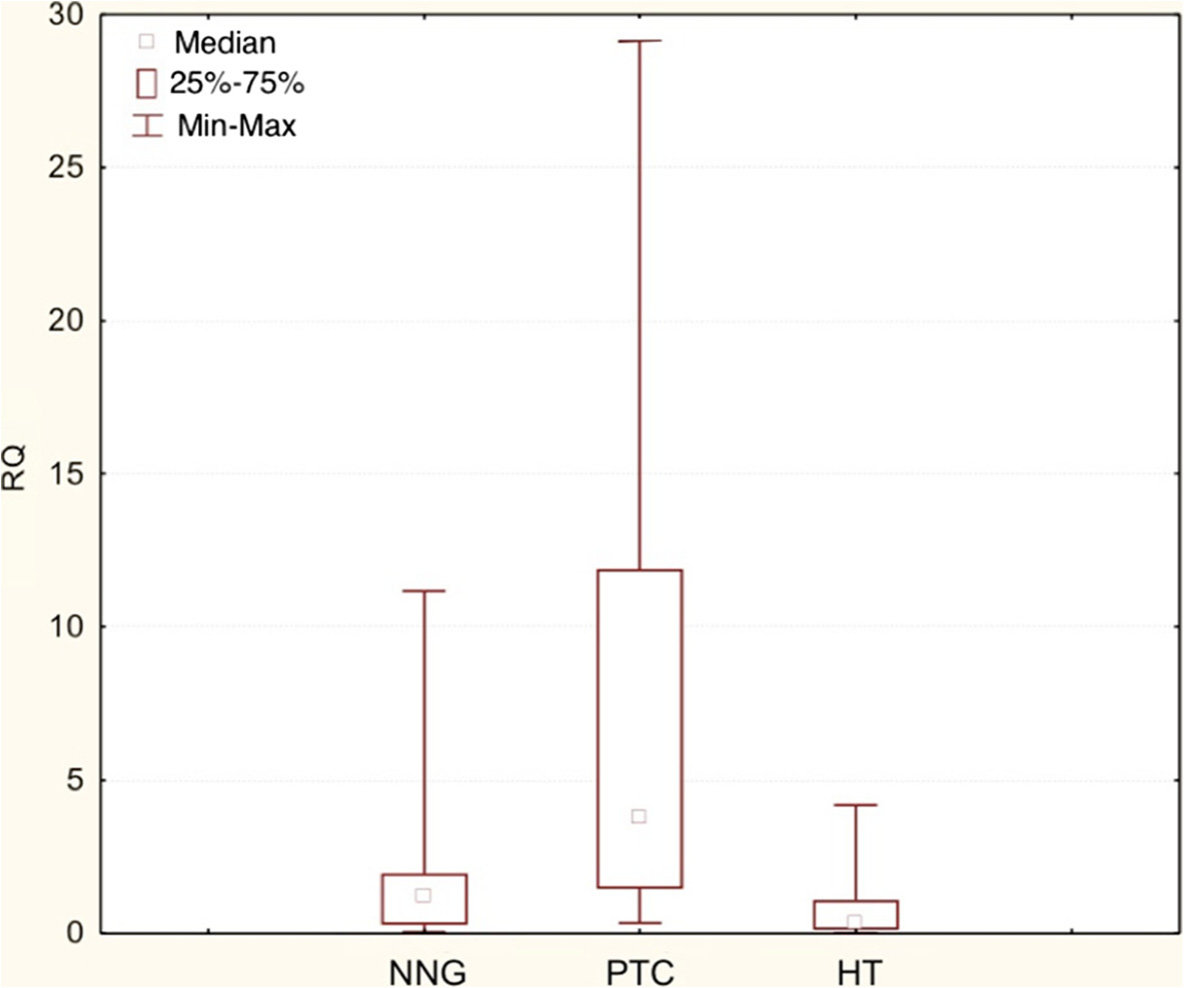
**Box-and-whisker plots representing the** ***COX-2*** **gene expression by quantitative RT-PCR in NNG, PTC and HT groups (FNAB).** Results are calculated as RQ values. Whiskers represent mean ± SD (standard deviation) for particular groups. Boxes represent mean ± SEM (standard error of mean). The results were statistically analyzed using the Kruskal-Wallis test (p < 0.0001).

*COX-2* mRNA expression was significantly higher in PTC when compared with HT and NNG groups (FNAB washouts) (p < 0.0001). The box-and-whisker plot diagrams, representing the expression levels of *COX-2* (mean and median values) in FNAB washouts, are presented in Figures 1 and 2, respectively.

**Figure 3:**
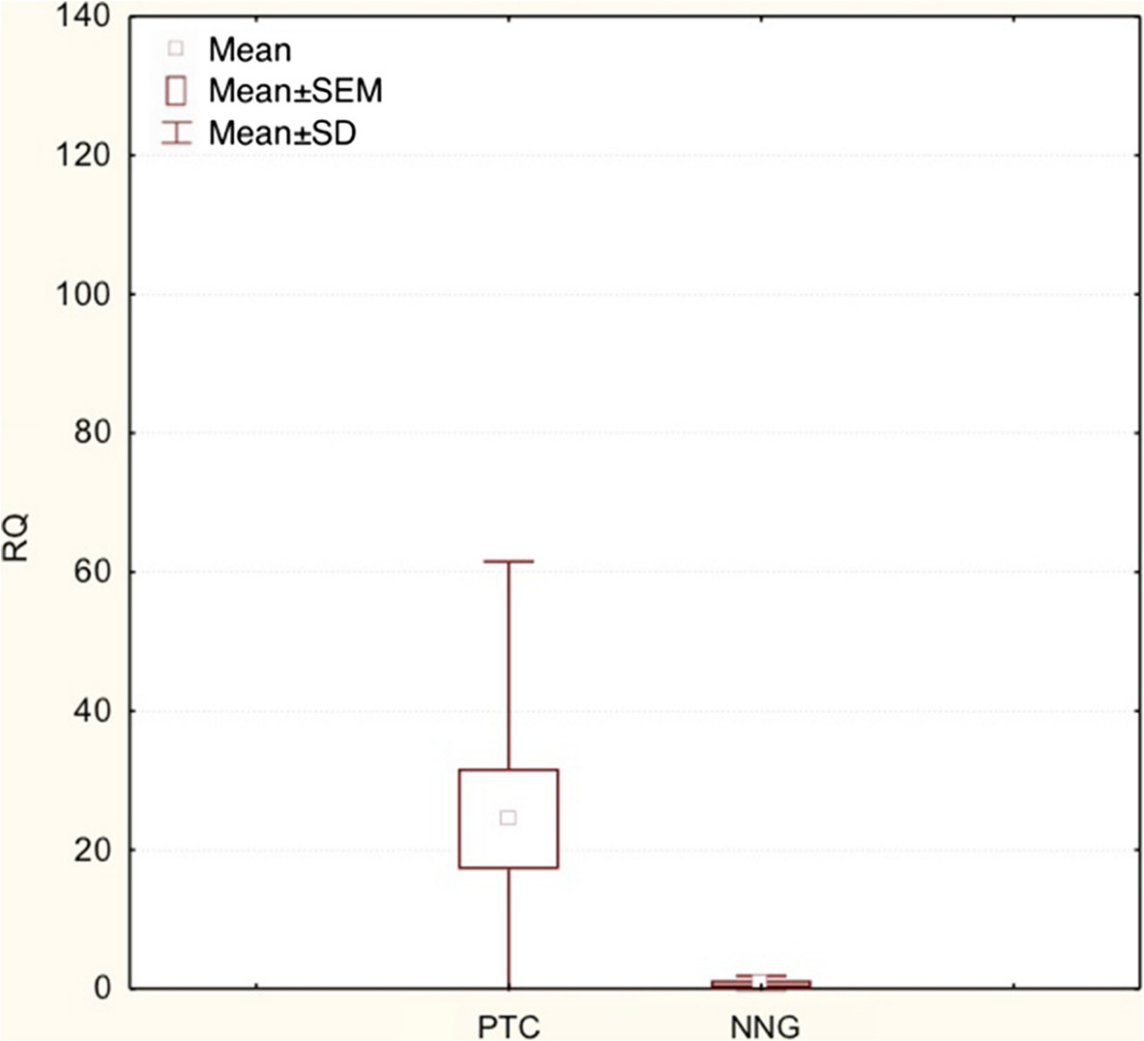
**Box-and-whisker plots representing the** ***COX-2*** **gene expression by quantitative RT-PCR in PTC, NNG groups (post-surgical tissues).** Results are calculated as RQ values. Whiskers represent mean ± SD (standard deviation) for particular groups. Boxes represent lower quartile and upper quartile. The results were statistically analyzed, using Man-Whitney U test (p < 0.05).

**Figure 4:**
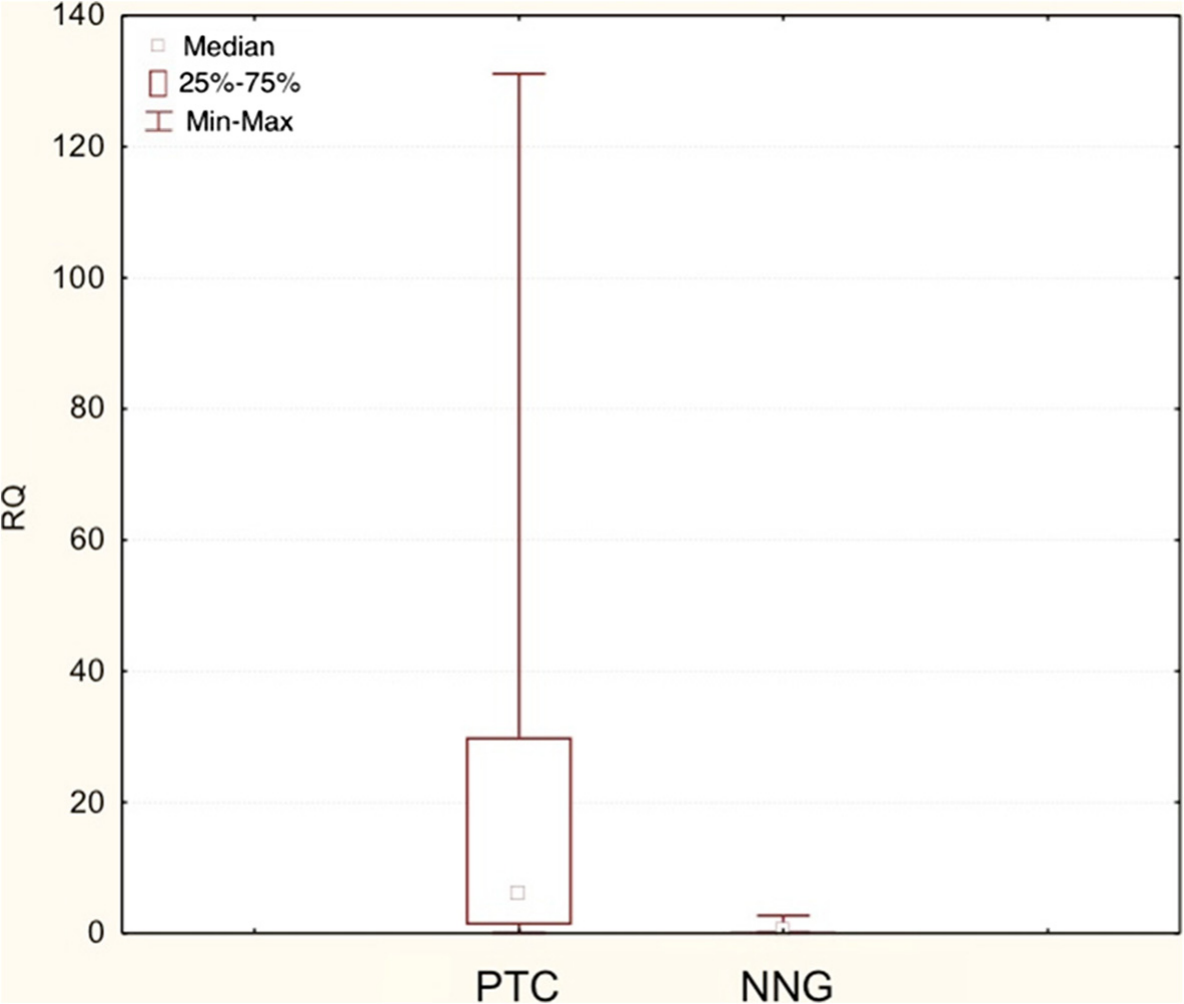
**Box-and-whisker plots representing the** ***COX-2*** **gene expression by quantitative RT-PCR in PTC, NNG groups (post-surgical tissues).** Results are calculated as RQ values. Whiskers represent median and minimum and maximum values for particular groups. Boxes represent lower quartile and upper quartile. The results were statistically analyzed, using Kruskal–Wallis test (p < 0.05).

*COX-2* expression was significantly higher in PTC when compared to NNG group (postsurgical material) (p < 0.05) (Figures 3 and 4).

**Figure 5:**
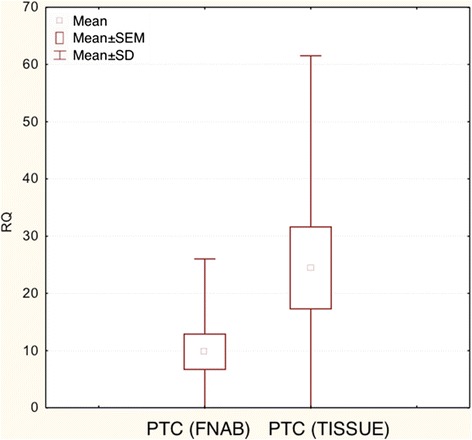
**Box-and-whisker plots, representing the expression of** ***COX-2*** **gene in the studied groups (FNAB, post-surgical tissues).** The results are calculated as RQ values. Whiskers represent mean ± SD (standard deviation) for particular groups. Boxes represent mean ± SEM (standard error of mean). The results were statistically analyzed, using Man-Whitney U test (p < 0.05).

**Figure 6:**
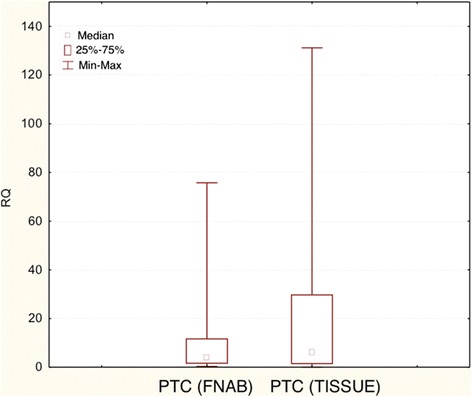
**Box-and-whisker plots, representing the expression of** ***COX-2*** **gene in the studied groups (FNAB, post-surgical tissues).** The results are calculated as RQ values. Whiskers represent median and minimum and maximum values for particular groups. Boxes represent lower quartile and upper quartile. The results were statistically analyzed, using Man-Whitney U test (p < 0.05).

In both PTC and NNG no significant differences in *COX-2* gene expression levels were found in the material obtained from the FNAB, and in postoperative tissue material (Student’s t-test, p > 0.05 in each case). The box-and-whisker plot diagrams, representing the expression levels of *COX-2* (median and mean values) in FNAB and in postsurgical PTC material, are presented in Figures 5 and 6, respectively (data for NNG not shown).

There was no correlation between *COX-2* expression and anti-TPO antibodies levels in the HT group. Also, there were no correlations between *COX-2* expression and patient’s gender or age in both PTC and NNG groups. Moreover, correlation between *COX-2* gene expression level and pTNM staging in pT1a and pT2-T4 was not found (p > 0.05).

*COX-1* expression occurred in all the test samples but the recorded differences in the gene expression levels in malignant and benign lesions, both in biopsy washouts and in postoperative tissue, were - in contrast to *COX-2* - not statistically significant (data not shown).

## Discussion

The expression of mediators of inflammation in physiological conditions and their role in the development of neoplastic processes within the thyroid gland is not yet fully clarified.

There have been several reports on an increased expression of COX-2 in different thyroid neoplasms. These data suggest that the enzyme may play a role in the initiation or progression of malignant tumours in the thyroid [13]. It is worth noting that the majority of studies concern evaluation of the COX-2 activity by immunohistochemical approach, and only a few authors have used the molecular biology methods.

In previously published reports, COX-2 expression was found in tissue from patients with differentiated thyroid carcinoma, HT and NNG [13–18]. Immunohistochemical studies demonstrated a high level of COX-2 expression in both the PTC and HT but low or negligible in the NNG and normal thyroid follicular cells, suggesting a relationship between an autoimmune disease and the process of carcinogenesis.

The study of Lee et al. [19] has shown prominent expression of COX-2 in thyroiditis, as well as benign and malignant thyroid nodules but not in normal thyroid tissue. The authors also observed no correlation between severity of PTC with or without metastasis. Because of the similar intensity of COX-2 staining found in thyroiditis and thyroid nodules, the authors concluded only little probability that COX-2 expression is related to the progression of thyroid disease [19].

Specht et al. [20] revealed overexpression of *COX-2* in 8 out of 10 cases of thyroid cancer, specific to tumour cells but not to the surrounding stroma. They have shown that *COX-2* mRNA and protein were increased in malignant thyroid nodules, when compared with benign nodules and adjacent thyroid tissue.

In our study the *COX-2* gene expression level was significantly higher in cytological material collected from patients with PTC when compared to NNG and HT and significantly higher in the tissue material of PTC, comparing with NNG. In addition, in the present study the expression of *COX-2* in patients with HT proved to be comparable with the expression in NNG.

The recent study of other authors suggests that the high expression of *COX-2* and low expression of *KAI-1/CD82* are associated with increased tumour invasiveness [21]. The results of our research, similarly to previous literature report [22], do not indicate a correlation between these parameters. However, failing to confirm the existence of such a correlation in our present investigation, can result from a relatively small number of cases comprised in the study. It should also be noted that in our research correlation analysis related only to the size of the tumour in TNM scale but without metastases, as there were none in our group of patients.

Our results together with the previously published studies [14,23,24] confirmed the validity of genetic analysis in FNAB derived material. In our present study, the results of *COX-2* expression in tissue and FNAB washouts material were comparable. This observation confirms the significance of genetic analysis of this material being usually not abundant (rather scarce), but sufficient to analyze by means of ultrasensitive methods.

In the published medical reports we have not met attempts to link the *COX-2* gene expression and anti-TPO levels. Only in one study, the presence of specific autoantibodies itself rather than their level (concentration), proved to be crucial [25]. In our research, we have shown no relationship between the anti-TPO concentrations and *COX-2* gene expression levels of the patients studied.

In the available literature, we have not met so far any attempt to link the *COX-2* expression with patient’s gender. Our study, however, has shown no statistically significant relationship between the *COX-2* expression level and patient’s gender, although the significant disparity between the number of men (28) and women (143) in the study group should be noted.

So far, only a few authors have tried to evaluate the *COX-1* expression in thyroid diseases. In clinical study of Lee et al. [26], *COX-1* expression was low and similar in different thyroid conditions. Results obtained by us in the present study proved to be consistent with the aforementioned report. *COX-1* expression occurred in all the test samples but the differences in gene expression levels in malignant and benign lesions in both cytological and postoperative materials - in contrast to *COX-2*, were not statistically significant.

## Conclusions

The present study confirms the validity of genetic analysis of material collected from FNAB. The new methods of molecular biology are of a such sensitivity that they allow for the precise assessment of gene expression, even if a small amount of genetic material is accessible.

The obtained results suggest that *COX-2* gene does not participate in mechanisms involved in molecular association of HT with PTC. However, in case of PTC itself, COX-2 expression is not a sufficient factor to become a marker of ongoing cancer in the thyroid, nevertheless, it may play a role in neoplastic transformation.

Epidemiological and histological studies indicate a relationship between thyroid neoplasms and Hashimoto disease, between COX-2 activity and carcinogenesis, but also the further need to search for connecting factors and randomized controlled trials to assess the actual frequency of co-occurrence of these disease entities and their molecular basis.
